# Group decision making in the analytic hierarchy process by hesitant fuzzy numbers

**DOI:** 10.1038/s41598-023-49076-3

**Published:** 2023-12-10

**Authors:** Mahdi Ranjbar, Sohrab Effati

**Affiliations:** 1 Applied Mathematics, Ferdowsi University of Mashhad, Mashhad, Iran; 2 Member of the center of Excellent on Soft Computing and Intelligent Information Processing, Ferdowsi University of Mashhad, Mashhad, Iran

**Keywords:** Applied mathematics, Computational science

## Abstract

Due to the increasing complexity of decision problems, many managers employ multiple experts to reach a good decision in a group decision making. Now, if there is ambiguity in the evaluation of experts, the use of fuzzy numbers can be useful for each expert. In these situations, the use of hesitant fuzzy numbers (HFNs) which consists of several fuzzy numbers with special conditions can be suggested. HFNs are as an extension of the fuzzy numbers to take a better determining the membership functions of the parameters by several experts. Because of simple and fast calculations, in this paper, we use triangular HFNs in the pairwise comparison matrix of analytic hierarchy process by opinions of a group of decision makers in a hesitant fuzzy environment. We define consistency of the hesitant fuzzy pairwise comparison matrix and use the arithmetic operations on the HFNs and a new method of comparing HFNs to get the hesitant fuzzy performance score. By using score function to hesitant fuzzy score we can get a final score for alternatives. Finally, a practical example is provided to show the the effectiveness of this study. The obtained results from this paper show that new method can get a better answer by keeping the experts’ opinions in the process of solving the problem.

## Introduction

It is difficult for an expert to be able to consider all aspects of a decision-making problem. Therefore, group decision-making would often be preferred and would generate more benefits than individual decision-making. The relationships among the decision makers are important factors that affect on group decision-making process^1^. Also, if they are like-minded, they are aligned in choosing their opinions, but they may have hesitance in choosing the membership function as a fuzzy number in different forms. In most research articles on group decision-making, the opinions of different decision makers are aggregated, which causes the loss of some information. In such a situation, using a new approach can be useful. In this article, we try to solve this problem by considering the extension of fuzzy numbers and using the existing arithmetic operations on them.

In the theory of decision making, the analytic hierarchy process (AHP) is a structured technique for organizing and analyzing complex decisions. It was developed by Saaty^2^, which the experts usually provide crisp values for decisions over paired comparisons of alternatives with respect to a criterion. If the experts are uncertain on the decisions, this uncertainty can be measured by intervals^3^. In uncertain situations, the decisions can also be represented by fuzzy values. As a popular methodology for confronting with uncertainty, the fuzzy logic combined with AHP, more commonly known as fuzzy AHP (FAHP), has found more applications in recent years^4^. Laarhoven and Pedrycz^5^ presented a fuzzy version of AHP method. Buckley used fuzzy priorities of comparison ratios in place of exact ratios^6^. Chang introduced a new approach for FAHP with using triangular fuzzy numbers in pairwise comparison scale^7^. Cheng presented a new approach for evaluating naval tactical missile systems depending by the FAHP^8^. Chan and Kumar used fuzzy extended AHP-based approach to global supplier development considering risk factors. Huang et al. presented a FAHP method and utilize crisp judgment matrix to evaluate subjective expert judgments made by the technical committee of the Industrial Technology Development Program in Taiwan^9^. Tang provided an efficient budget allocation method using FAHP for businesses^10^. Das et al. focused on performance evaluation and ranking of seven Indian institute of technology in respect to stakeholders’ preference using an integrated model consisting of FAHP and compressed proportional assessment methods^11^. Deng applied a FAHP approach for tackling qualitative multi criteria analysis problems^12^. Cheng et al. considered attack helicopters based on linguistic variables by a FAHP method^13^. Leung and Cao proposed a fuzzy consistency of a tolerance deviation in the FAHP method^14^. Karczmarek et al. developed FAHP in a graphical approach.

Other extensions of FAHP have been also developed in the literature such as type-2 AHP, intuitionistic FAHP (IFAHP), neutrosophic AHP (NAHP) and hesitant FAHP (HFAHP). Kahraman et al. integrated type-2 fuzzy sets with AHP^15^. Sari et al. applied interval type-2 fuzzy AHP into warehouse location selection problem^16^. Oztaysi used interval type-2 fuzzy AHP method into group decision making problem for information systems selection problem^17^. Sadiq and Tesfamariam introduced an environmental decision-making under uncertainty using IFAHP^18^. Lazim and Liana proposed a new IFAHP method characterised by new preference scale of pair-wise comparison matrix measurement^19^. Abdel-Basset et al. given an overview of the AHP in neutrosophic environment^20^. Slamaa et al. studied comparative analysis of AHP, FAHP and NAHP based on multi-criteria decision making^21^. Navarro et al. proposed a NAHP completion methodology to reduce the number of judgments required to be emitted by the decision maker^22^. Vafadarnikjoo et al. analyzed the barriers to blockchain technology adoption in manufacturing supply chains using the NAHP^23^. Verma et al. proposed a NAHP approach for budget constrained reliability allocation^24^.

Fuzzy statistics and neutrosophic statistic can have applications in decision-making problems. In fuzzy statistics we use fuzzily perceived or linguistic values often in the form triangular/trapezoidal fuzzy numbers^25^. Neutrosophic statistics is a generalization of traditional statistics that is used to analyze uncertain, unclear, vague, and incomplete data^26^. For example Foroozesh et al. studied a new soft computing approach based on multi-attributes decision analysis, group decision making and fuzzy possibilistic statistical modeling for sustainable supplier selection problem^27^. Gurmani et al. presented an interaction and feedback mechanism-based group decision-making for emergency medical supplies supplier selection using T-spherical fuzzy information^28^. AlAita et al. introduced a new approach is proposed using neutrosophic statistics to analyze split-plot and split-block designs. By such an approach neutrosophic hypothesis is formulated and a decision rule is suggested^29^. Aslam proposed a new attribute sampling plan using neutrosophic statistical interval method^30^. Afzal et al. proposed a neutrosophic statistical approach for the analysis of resistance of conducting material depending on the temperature variance^31^. Nagarajan et al. introduced a novel approach based on neutrosophic Bonferroni mean operator of trapezoidal and triangular neutrosophic interval environments in multi-attribute group decision making^32^.

Torra^33^ introduced hesitant fuzzy sets (HFSs) that are one of major supportive tools for multi-criteria decision-making techniques for dealing with the situations where experts have hesitancy in providing their preferences over objects. Hesitancy is a common phenomenon in the process of human reasoning, especially in operation research and decision making problems. For example, Rodriguez et a. used hesitant fuzzy linguistic term sets for decision making problems^34^. In order to better solve the multi-attribute group decision making problems, Xu and Zhang extended the Technique for Order Performance by Similarity to Ideal Solution (TOPSIS) method to the hesitant fuzzy environment^35^. Xia et al. defined some hesitant fuzzy aggregation operators and applied them in group decision making^36^. Wang et al. improved TOPSIS model in the q-rung orthopair hesitant fuzzy environment^37^. Chen and Xu proposed the hesitant fuzzy ELECTRE II^38^. Mahmoudi et al. applied hesitant fuzzy elements to PROMETHEE method and established hesitant fuzzy PROMETHEE^39^. Lin et al. studied on decision making with probabilistic hesitant fuzzy information^40^. Zhang et al. proposed the hesitant fuzzy linguistic linear programming technique for multidimensional analysis of preference (LINMAP) method based on the interval programming model^41^. Xu et al. developed a new method called hesitant fuzzy LINMAP, which combines the hesitant fuzzy linguistic term sets with LINMAP method^42^. Liu et al. extended the LINMAP to accommodate hesitant fuzzy environment and propose a new approach to solve the multi-attribute decision making problems with hesitant fuzzy information, then an integrated method that combines the LINMAP and TOPSIS is developed^43^. Tang et al. presented a group decision making with interval linguistic hesitant fuzzy preference relations^44^. Ranjbar et al. presented a new approach for fuzzy classification in the hesitant environments by decision-making process^45^. Rouhbakhsh et al. used HFSs in multiobjective programming problems^46^. Molinera et al. presented a novel group decision making method for dynamic contexts with a high number of decision alternatives using HFSs^47^. Xu and Zhang presented an overview on the applications of the HFSs in group decision-making^48^. Wan et al. developed a hesitant fuzzy PROMETHEE for multi-criteria group decision-making and applies to green supplier selection^49^. Ranjbar and Effati used HFSs in mathematical programming problems^50^. Zheng et al. proposed a new hesitant fuzzy linguistic method to deal with issues when a lot of decision makers provide hesitant and uncertain preference information in the decision-making process^51^. Wu et al. used hesitant fuzzy preference relations in the graph model for conflict resolution^52^. Deli and Karaaslan proposed a decision-making method to solve the multi-criteria decision-making problems in which criteria values take the form of generalized trapezoidal hesitant fuzzy information^53^. Keikha generalized hesitant fuzzy numbers and their application in solving multi-attribute decision-making problems^54^. Ranjbar et al. developed the hesitant fuzzy arithmetic and ordering method on HFNs, then use them on simple additive weighting (SAW) method based on the extension of Bonissone’s approach in the hesitant fuzzy environment^55^. Also they used HFNs in optimization problems^56,57^. Ashraf et al. proposed a model for emergency supply management under extended EDAS method and spherical hesitant fuzzy soft aggregation information^58^. Jeon et al. proposed an innovative probabilistic hesitant fuzzy elements based on multi-criteria decision-making perspective^59^.

Among these, the HFAHP is one of the methods that is widely used in the literature. At first Zhu and Xu used hesitant judgments in analytic hierarchy process-group decision making^60^. Mousavi et al. introduced the HFAHP method^61^. Then, Oztaysi et al. extended an HFAHP method with linguistic evaluations of several experts^62^. Zhu et al. developed a hesitant AHP method as an extension of traditional AHP^63^. Mi et al. designed the framework of the hesitant fuzzy linguistic AHP^64^. Singh et al. proposed a new method using AHP by hesitant probabilistic fuzzy linguistic set^65^. Also, many applications of HFAHP presented in the literature. For example, Cevik Onar et al. applied hesitant fuzzy linguistic term sets (HFLTSs) based AHP and TOPSIS methods^66^. Kahraman et al. used a hesitant fuzzy linguistic AHP method for the selection among business to customer firms^67^. Acar et al. to overcome the observed hesitancy in decision makers’ preferences used hesitant fuzzy AHP to evaluate sustainability of the selected hydrogen production methods^68^. Camci et al. introduced an HFAHP based multi-criteria decision making system for computer numerical control router selection^69^. Tuysuz and Simsek have also benefited HFLTS based on HFAHP in order to evaluate performance of the logistics firm which has 1000 branches in Turkey^70^. Buyukozkan and Guler proposed an supply chain analytics tool evaluation model by using HFLTS and AHP method^71^. Samanlioglu et al. applied HFAHP to measurement of the COVID-19 pandemic intervention strategies^72^. Candan and Toklu solved the most appropriate location problem for the solar power plant by HFAHP method^73^. Batur Sir and Sir used an HFLTS in the AHP and VIKOR method to treat the pain symptoms observed in COVID-19 patients^74^. Candan and Cengiz determined solar power plant location using HFAHP method^73^.

In most of these studies for HFAHP method used HFSs or hesitant fuzzy linguistic term sets to select elements of hesitant fuzzy pairwise comparison matrix (HFPCM), which usually solve these problems by aggregation the opinions of decision makers. In this paper, we want to use these elements on a special type of the HFNs, which creates a new form for using AHP method in a hesitant fuzzy environment. Then, the required definitions and theorems have been prepared and a new algorithm introduced to rank of alternatives by AHP method in these conditions. One of the advantages of this approach is that the HFNs are effective on arithmetic operations, similar to the fuzzy numbers in fuzzy mathematics, that cause to reduce the volume of calculations and to apply the expertise of decision makers in all problem-solving processes.

The remainder of this paper has been formed as follows: In section "Preliminaries", we provide some needed definitions and notions. In section "Algorithm of new approach for HFAHP", we propose an algorithm to solve HFAHP method. In section "Illustration", one example to illustrate of the proposed algorithm is provided.The comparative analysis is done in section "Comparative analysis". A discussion is given in section "Discussion". Finally, some conclusions and recommendations for future research are discussed in section "Conclusion".

## Preliminaries

### Definition 1

^75^ If *S* is a collection of objects denoted by *s*, then a fuzzy set ${\tilde{F}}$ in *S* is a set of ordered pairs

$$\begin{aligned} {\tilde{F}}=\lbrace \big ( s,\mu _{{\tilde{F}}}(s) \big )\,\, \vert \,\, s \in S \rbrace , \end{aligned}$$

which $\mu _{{\tilde{F}}}(s)$ is entitled the membership function of *x* in ${\tilde{F}}$.

Fuzzy numbers are a type of fuzzy sets that on the set ${\mathbb {R}}$ under special conditions are defined. Triangular and trapezoidal fuzzy numbers are often used to sake of computational efficiency. A trapezoidal fuzzy number is a fuzzy number represented with quaternary notation as ${\tilde{F}} = (f_1, f_2, f_3, f_4)$, this representation is interpreted as membership function as follows:

$$\begin{aligned} \mu _{{\tilde{F}}}(s)={\left\{ \begin{array}{ll} 0 \,\,\,\,\,\,\,\,\,\,\,\,\,\,\,\,\,\,\,\,\, \text {for all} \,\, s \in (-\infty ,f_1],\\ \frac{s-f_1}{f_2-f_1} \,\,\,\,\,\,\,\,\,\,\,\, \text {for all} \,\, s \in [f_1,f_2],\\ 1 \,\,\,\,\,\,\,\,\,\,\,\,\,\,\,\,\,\,\,\,\, \text {for all} \,\, s \in [f_2,f_3],\\ \frac{f_4-s}{f_4-f_3} \,\,\,\,\,\,\,\,\,\,\,\, \text {for all} \,\, s \in [f_3,f_4],\\ 0 \,\,\,\,\,\,\,\,\,\,\,\,\,\,\,\,\,\,\,\,\, \text {for all} \,\, s \in [f_4,\infty ).\\ \end{array}\right. } \end{aligned}$$

Also, if in the quaternary notation $(f_1, f_2, f_3, f_4)$ we have $f_2=f_3$, then we can be represented it by the ternary notation $(f_1, f_2, f_4)$ as a triangular fuzzy number (TFN).

In the next definition, we introduce the HFSs.

### Definition 2

^33^ Let *Y* be a reference set which its objects defined by *y*; then the HFS $\tilde{{\tilde{F}}}$ on *Y* is defined as a set of ordered pairs as follows:

$$\begin{aligned} \tilde{{\tilde{F}}}=\lbrace \big ( y,h_{\tilde{{\tilde{F}}}}(y) \big )\,\, \vert \,\, y \in Y \rbrace , \end{aligned}$$

where $h_{\tilde{{\tilde{F}}}}(y)=\lbrace f_1, \ldots f_{l( y )} \rbrace$ with $l( y )=\vert h_{\tilde{{\tilde{F}}}}(y) \vert$ is the possible membership degrees of the element $y \in Y$ to the set $\tilde{{\tilde{F}}}$. For convenience, $h_{\tilde{{\tilde{F}}}}(y)$ is named a hesitant fuzzy element (HFE).

### Definition 3

^76^ For an HFE $h_{\tilde{{\tilde{F}}}}(y)$, $S(h_{\tilde{{\tilde{F}}}}(y)) = \sum _{j=1}^{l(y)}\frac{f_j}{l(y)}$ is named the score function of $h_{\tilde{{\tilde{F}}}}(y)$, where *l*(*y*) is the cardinality of $h_{\tilde{{\tilde{F}}}}(y)$.

Some operations on two HFE $h_1$ and $h_2$ and $\lambda \in {\mathbb {R}}^+$ are defined in^76^ as follows:

- $$(h_{1})^\lambda =\bigcup _{f_1 \in h_{1}} \lbrace f_1^\lambda \rbrace .$$
- $$\lambda (h_{1})=\bigcup _{f_1 \in h_{1}} \lbrace 1-(1-f_1)^\lambda \rbrace .$$
- $$h_{1} \oplus h_{2}=\bigcup _{f_1 \in h_{1}, f_2 \in h_{2}} \lbrace f_1 + f_2 - f_1 f_2 \rbrace .$$
- $$h_{1} \otimes h_{2}=\bigcup _{f_1 \in h_{1}, f_2 \in h_{2}} \lbrace f_1 f_2 \rbrace .$$

### Remark 1

^55^ $\tilde{{\tilde{F}}}=\int _{Y}\frac{h_{\tilde{{\tilde{F}}}}(y)}{y},$ denote an HFS, which *Y* is infinite and $h_{\tilde{{\tilde{F}}}}(y)=\lbrace \mu _{{\tilde{F}}^1}(y), \ldots \mu _{{\tilde{F}}^{l( y )}}(y) \rbrace$.

### Definition 4

^77^ A HFS $\tilde{{\tilde{U}}}$ on *Y* is defined uniformly HFS (UHFS) if there is a number *p* such that $l( y ) \le p$ for each $y \in Y$.

Characteristic of the each element of UHFS $\tilde{{\tilde{U}}}$ defined as $Char(h_{\tilde{{\tilde{U}}}})=max \lbrace l(y) : y \in Y\rbrace .$ Also, if *Y* is infinite, we express the UHFS $\tilde{{\tilde{U}}}$ with $\tilde{{\tilde{U}}}=\lbrace {\tilde{U}}^j, \rbrace _{j=1}^{p}$, while $l( y ) = p$ for all $y \in Y$.

In the following the definition of an HFN is introduced.

### Definition 5

^78^ Let $\tilde{{\tilde{E}}}$ be a UHFS as follows:

$$\begin{aligned} \tilde{{\tilde{E}}}=\lbrace {\tilde{E}}^j \rbrace _{j=1}^{p}. \end{aligned}$$

Then we named it an HFN, if

1. $${\tilde{E}}^j \in {\mathbb {F}},$$    $$\forall j=1, \ldots , p$$.
2. $$\bigcap _{j=1}^p \, {\tilde{E}}^j_{1} \ne \emptyset$$.

where ${\tilde{E}}^j_{1}$ is an 1-cut for the *j*th ($j=1, \ldots , p$) element of the UHFS $\tilde{{\tilde{E}}}$ and ${\mathbb {F}}$ is space of fuzzy numbers.

**Figure 1:**
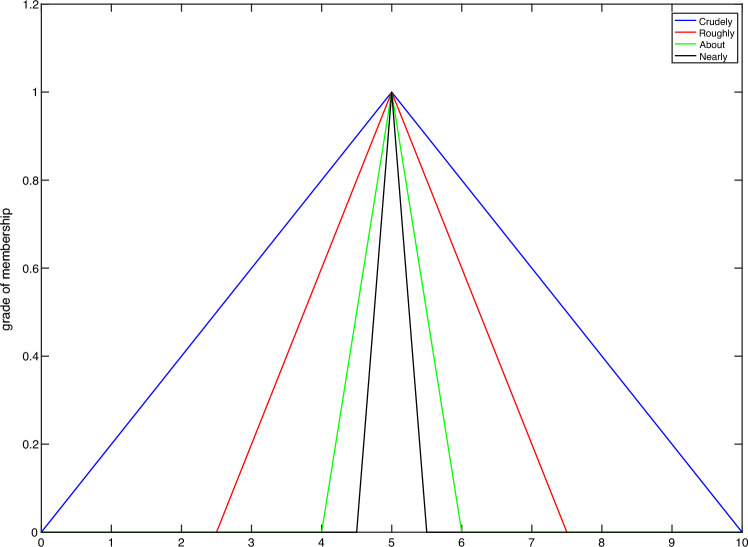
Various hedges for the fuzzy number ’${\tilde{5}}'$ by four decision makers.

The space of HFNs denoted with $\mathbb{H}\mathbb{F}$. One reason for using HFNs is that in some of decision-making problems, all experts agree on a fuzzy number as linguistic value for a attribute of the alternative, but disagree on the choice of the hedge term for that item. For example, when experts evaluate the 'Design’ of a car, linguistic labels like 'Good’, 'Fair’ and 'Weak’ are usually used. Let for label 'Fair’, all experts agree with the fuzzy number ’${\tilde{5}}$’, but there is a difference in determining its hedges. In such situations we propose to use of the HFNs. Figure 1 shows various hedges for the fuzzy number ’${\tilde{5}}$’ by four decision makers.

### Definition 6

We define a HFN $\tilde{{\tilde{T}}}=\lbrace {\tilde{T}}^j \rbrace _{j=1}^{p}$ to be a triangular HFN (THFN) if each fuzzy number ${\tilde{T}}^j$ for all $j=1, \ldots , p$ is a TFN as $(L_{T}^{j},T,U_{T}^{j})$, where $L_{T}^{j} \le T \le U_{T}^{j}$.

HFN $\tilde{{\tilde{5}}}$ in Fig. 1 is a THFN. In this paper, we use arithmetic operations that have been recently introduced in^55^ on HFNs for two HFNs $\tilde{{\tilde{G}}}$ and $\tilde{{\tilde{H}}}$ as follows:

1 $$\begin{aligned} \tilde{{\tilde{G}}} \mathop {*}\limits _{\tilde{\tilde{}}} \tilde{{\tilde{H}}}(z)=\bigcup _{j=1, \ldots , p} \bigg \lbrace \sup _{z=x*y} \big \lbrace \min \, \lbrace \mu _{{\tilde{G}}^{\sigma (j)}}(x), \mu _{{\tilde{H}}^{\sigma (j)}}(y) \rbrace \big \rbrace \bigg \rbrace \end{aligned}$$

for all $z \in {\mathbb {R}}$, and let $\sigma :(1, \ldots ,p) \rightarrow (1, \ldots ,p)$ be a permutation, where ${\tilde{G}}^{\sigma (j)}$ and ${\tilde{H}}^{\sigma (j)}$ are the *j*th smallest membership function in HFNs $\tilde{{\tilde{G}}}$ and $\tilde{{\tilde{H}}}$, respectively, that ordered by a ranking function as Yager index^79^.

Now, if $\tilde{{\tilde{T}}}_1=\lbrace {\tilde{T}}_1^{\sigma (1)}, \cdots , {\tilde{T}}_1^{\sigma (p)} \rbrace$ and $\tilde{{\tilde{T}}}_2=\lbrace {\tilde{T}}_2^{\sigma (1)}, \cdots , {\tilde{T}}_2^{\sigma (p)} \rbrace$ be two THFNs in Definition 2.6, where ${\tilde{T}}_1^{\sigma (l)}=(L_{T_1}^{\sigma (l)},T_1,U_{T_1}^{\sigma (l)}) > 0$ and ${\tilde{T}}_2^{\sigma (l)}=(L_{T_2}^{\sigma (l)},T_2,U_{T_2}^{\sigma (l)}) > 0$ for all $l \in \lbrace 1, \ldots , p \rbrace$, we have:

1. $$\tilde{{\tilde{T}}}_1 \mathop {\oplus }\limits _{\tilde{\tilde{}}} \tilde{{\tilde{T}}}_2=\tilde{{\tilde{T}}}_3=\lbrace {\tilde{T}}_3^{\sigma (l)} \rbrace _{l=1}^{p}$$,
  where
  $$\begin{aligned} {\tilde{T}}_3^{\sigma (l)}=(L_{T_1}^{\sigma (l)} + L_{T_2}^{\sigma (l)}, T_1 + T_2, U_{T_1}^{\sigma (l)} + U_{T_2}^{\sigma (l)}) \end{aligned}$$
  for each $$l= 1, \ldots , p$$.
2. $$\tilde{{\tilde{T}}}_1 \mathop {\otimes }\limits _{\tilde{\tilde{}}} \tilde{{\tilde{T}}}_2\approx \tilde{{\tilde{T}}}_4 =\lbrace {\tilde{T}}_4^{\sigma (l)} \rbrace _{l=1}^{p}$$,
  where
  $$\begin{aligned} {\tilde{T}}_4^{\sigma (l)}=(L_{T_1}^{\sigma (l)}L_{T_2}^{\sigma (l)}, T_1T_2, U_{T_1}^{\sigma (l)}U_{T_2}^{\sigma (l)}) \end{aligned}$$
  for each $$l= 1, \ldots , p$$.
3. $$\lambda \tilde{{\tilde{T}}}_1=\tilde{{\tilde{T}}}_5=\lbrace {\tilde{T}}_5^{\sigma (l)} \rbrace _{l=1}^{p}$$,
  where
  $$\begin{aligned} {\tilde{T}}_5^{\sigma (l)}=(\lambda L_{T_1}^{\sigma (l)}, \lambda T_1, \lambda U_{T_1}^{\sigma (l)}) \end{aligned}$$
  for each $l= 1, \ldots , p$ and $\lambda > 0, \lambda \in {\mathbb {R}}$.
4. $$(\tilde{{\tilde{T}}}_1)^{-1}\approx \tilde{{\tilde{T}}}_6 =\lbrace {\tilde{T}}_6^{\overline{\sigma }(l)} \rbrace _{l=1}^{p}$$,
  where
  $$\begin{aligned} {\tilde{T}}_6^{\overline{\sigma }(l)}=(\dfrac{1}{U_{T_1}^{\overline{\sigma }(l)}}, \dfrac{1}{T_1}, \dfrac{1}{L_{T_1}^{\overline{\sigma }(l)}}) \end{aligned}$$
  for each $$l= 1, \ldots , p$$.

It should be noted that, for more convenience in calculations in practice, the HFN $\tilde{{\tilde{T}}}_1 \mathop {\otimes }\limits _{\tilde{\tilde{}}} \tilde{{\tilde{T}}}_2$ which its elements are not necessarily TFNs and can be approximated by the TFNs using the hypothesis of left and right divergence^80^ and it call an approximation of the given HFN.

For definition of consistency in hesitant fuzzy pairwise comparison matrix (HFPCM), we need to introduce a method of comparing HFNs. Thus, by extension principle on HFSs we give the definition as follows.

### Definition 7

Let $\tilde{{\tilde{G}}}=\lbrace {\tilde{G}}^{\sigma (l)} \rbrace _{l=1}^{p}$ and $\tilde{{\tilde{H}}}=\lbrace {\tilde{H}}^{\sigma (l)} \rbrace _{l=1}^{p}$ be two HFNs, the hesitant degree of possibility of $\tilde{{\tilde{G}}} \ge \tilde{{\tilde{H}}}$ define as follows

2 $$\begin{aligned} h(\tilde{{\tilde{G}}} \ge \tilde{{\tilde{H}}})=\bigcup _{l=1, \ldots , p} \bigg \lbrace \sup _{s\ge t} \big \lbrace \min \, \lbrace \mu _{{\tilde{G}}^{\sigma (l)}}(s), \mu _{{\tilde{H}}^{\sigma (l)}}(t) \rbrace \big \rbrace \bigg \rbrace \end{aligned}$$

In Definition 7, if $\tilde{{\tilde{T}}}_1$ and $\tilde{{\tilde{T}}}_2$ are two THFNs and $T_1 \ge T_2$, then we have:

$$\begin{aligned} h(\tilde{{\tilde{T}}}_1 \ge \tilde{{\tilde{T}}}_2)=\lbrace 1, \ldots ,1 \rbrace \end{aligned}$$

and

$$\begin{aligned} h(\tilde{{\tilde{T}}}_2 \ge \tilde{{\tilde{T}}}_1)=\lbrace h^{\sigma (1)}(\tilde{{\tilde{T}}}_2 \ge \tilde{{\tilde{T}}}_1), \ldots , h^{\sigma (p)}(\tilde{{\tilde{T}}}_2 \ge \tilde{{\tilde{T}}}_1)\rbrace \end{aligned}$$

where $h^{\sigma (l)}(\tilde{{\tilde{T}}}_2 \ge \tilde{{\tilde{T}}}_1)$ for each $l \in \lbrace 1, \ldots , p\rbrace$ is the highest intersection between $\mu _{{\tilde{T}}_1^{\sigma (l)}}$ and $\mu _{{\tilde{T}}_2^{\sigma (l)}}$ in Dubios and Prade (1980) as follows:

$$\begin{aligned} h^{\sigma (l)}(\tilde{{\tilde{T}}}_2 \ge \tilde{{\tilde{T}}}_1)=\dfrac{L_{T_1}^{l} - U_{T_2}^{l}}{(T_2 - U_{T_2}^{l})-(T_1 - L_{T_1}^{l})}. \end{aligned}$$

### Example 1

Let $\tilde{{\tilde{3}}}=\lbrace (2,3,4), (2,3,6) \rbrace$ and $\tilde{{\tilde{4}}}=\lbrace (1,4,5), (2,4,6) \rbrace$ are two THFN. The hesitant degree of possibility of $\tilde{{\tilde{4}}} \ge \tilde{{\tilde{3}}}$ is as $h(\tilde{{\tilde{4}}} \ge \tilde{{\tilde{3}}})=\lbrace 1, 1 \rbrace$. Also, the hesitant degree of possibility of $\tilde{{\tilde{3}}} \ge \tilde{{\tilde{4}}}$ is as $h(\tilde{{\tilde{3}}} \ge \tilde{{\tilde{4}}})=\lbrace 0.75, 0.8 \rbrace$.

### Definition 8

Let $\tilde{{\tilde{G}}}=\lbrace {\tilde{G}}^{\sigma (l)} \rbrace _{l=1}^{p}$ and $\tilde{{\tilde{H}}}=\lbrace {\tilde{H}}^{\sigma (l)} \rbrace _{l=1}^{p}$ be two HFNs, We will say that $\tilde{{\tilde{G}}}$ is greater than $\tilde{{\tilde{H}}}$, written $\tilde{{\tilde{G}}} > \tilde{{\tilde{H}}}$, if

1. $$h(\tilde{{\tilde{G}}} \ge \tilde{{\tilde{H}}})=\lbrace 1, \ldots ,1 \rbrace$$.
2. $\forall l \in \lbrace 1, \ldots , p\rbrace$ we have $h^{\sigma (1)}(\tilde{{\tilde{H}}} \ge \tilde{{\tilde{G}}}) < \theta$, where $0 < \theta \le 1$.

According to Definition 8, in Example 1$\tilde{{\tilde{4}}} > \tilde{{\tilde{3}}}$.

### Definition 9

Let $\tilde{{\tilde{G}}}=\lbrace {\tilde{G}}^{\sigma (l)} \rbrace _{l=1}^{p}$ and $\tilde{{\tilde{H}}}=\lbrace {\tilde{H}}^{\sigma (l)} \rbrace _{l=1}^{p}$ be two HFNs, We will say that $\tilde{{\tilde{G}}}$ and $\tilde{{\tilde{H}}}$ are approximately equal which is written as $\tilde{{\tilde{G}}} \approxeq \tilde{{\tilde{H}}}$, if $\tilde{{\tilde{G}}}$ is not greater than $\tilde{{\tilde{H}}}$ and $\tilde{{\tilde{H}}}$ is not greater than $\tilde{{\tilde{G}}}$.

The crisp pairwise comparisons matrix ${\textbf {A}}=[a_{ij}]_{m \times m}$ consistent if only if $a_{ij} =a_{ik}a_{kj}$ for all *i*, *j* and *k*. Based on Definition 9 we define consistency for HFPCM $\tilde{{\tilde{A}}}$ as follows.

### Definition 10

An HFPCM as hesitant fuzzy positive symmetric matrix $\tilde{\tilde{{\textbf {A}}}}=[\tilde{{\tilde{a}}}_{ij}]_{m \times m}$ with HFNs is consistent if and only if $\tilde{{\tilde{a}}}_{ik} \mathop {\otimes }\limits _{\tilde{\tilde{}}} \tilde{{\tilde{a}}}_{kj} \approxeq \tilde{{\tilde{a}}}_{ij}$.

### Theorem 1

Let $\tilde{\tilde{{\textbf {B}}}}=[\tilde{{\tilde{T}}}_{ij}]_{m \times m}$ is a hesitant fuzzy positive symmetric matrix, where $\tilde{{\tilde{T}}}_{ij}=\lbrace {\tilde{T}}_{ij}^{\sigma (1)}, \ldots , {\tilde{T}}_{ij}^{\sigma (p)} \rbrace$ are THFNs for all $i=1, \cdots ,m, j=1, \cdots ,m$. If ${\textbf {B}}=[T_{ij}]$ is consistent, then $\tilde{\tilde{{\textbf {B}}}}$ is consistent.

### Proof

According to Theorem 2.1 in^6^, for each $l=1, \ldots , p$ we have

3 $$\begin{aligned} h^{\sigma (l)}({\tilde{T}}_{ik}^{\sigma (1)} \tilde{*} {\tilde{T}}_{kj}^{\sigma (1)} \ge {\tilde{T}}_{ij}^{\sigma (1)})=1 \end{aligned}$$

and

4 $$\begin{aligned} h^{\sigma (l)}({\tilde{T}}_{ij}^{\sigma (1)} \ge {\tilde{T}}_{ik}^{\sigma (1)} \tilde{*} {\tilde{T}}_{kj}^{\sigma (1)})=1, \end{aligned}$$

thus from (3) and (4) we have, $h(\tilde{{\tilde{T}}}_{ik} \mathop {\otimes }\limits _{\tilde{\tilde{}}} \tilde{{\tilde{T}}}_{kj} \ge \tilde{{\tilde{T}}}_{ij})=\lbrace 1, \ldots ,1 \rbrace$ and $h(\tilde{{\tilde{T}}}_{ij} \ge \tilde{{\tilde{T}}}_{ik} \mathop {\otimes }\limits _{\tilde{\tilde{}}} \tilde{{\tilde{T}}}_{kj})=\lbrace 1, \ldots ,1 \rbrace$. Hence, due to Definition 9$\tilde{{\tilde{T}}}_{ik} \mathop {\otimes }\limits _{\tilde{\tilde{}}} \tilde{{\tilde{T}}}_{kj} \approxeq \tilde{{\tilde{T}}}_{ij}$ and $\tilde{{\tilde{B}}}$ is consistent. $\square$

### Corollary 1

For the crisp pairwise comparisons matrix ${\textbf {B}}=[T_{ij}]$, the Consistency Ratio (*CR*) of ${\textbf {B}}$ is defined as

$$\begin{aligned} CR =\dfrac{\textit{consistency index}}{\textit{random index}} \end{aligned}$$

where the consistency index of ${\textbf {B}}$ is given by $\dfrac{\lambda _{max}-n}{n-1}$, which $\lambda _{max}$ is the largest eigenvalue of ${\textbf {B}}$ and the random index refers to the average consistency of randomly generated matrices of certain order, whose elements are chosen on 9-point scale. The solution to the AHP is acceptable only when the *CR* is less than or equal to 0.10 for all pairwise comparison matrices^2^. According to Theorem 1, for an HFAHP with HFPCMs $\lbrace \tilde{\tilde{{\textbf {B}}}}^l=[\tilde{{\tilde{T}}}_{ij}^l] \rbrace _{l=1}^t$, the solution to the HFAHP is acceptable only when the *CR* is less than or equal to 0.10 for all crisp pairwise comparison matrices as $\lbrace {\textbf {B}}^l=[T_{ij}^l] \rbrace _{l=1}^t$.

## Algorithm of new approach for HFAHP

In this algorithm for handling HFAHP method, we use the THFNs in pairwise comparison scale as a extension of the extent analysis method on FAHP in^7^. Based on, the new HFAHP method can be described as shown below in four algorithmic steps.

**Table 1:** The HFPCM of performance alternatives with the THFNs.

Alternative	$A_1$	$A_2$	$\ldots$	$A_m$
$A_1$	$\tilde{{\tilde{T}}}_{11}$	$\tilde{{\tilde{T}}}_{12}$	$\ldots$	$\tilde{{\tilde{T}}}_{1m}$
$A_2$	$\tilde{{\tilde{T}}}_{21}$	$\tilde{{\tilde{T}}}_{22}$	$\ldots$	$\tilde{{\tilde{T}}}_{2m}$
$\vdots$	$\vdots$	$\vdots$	$\ddots$	$\vdots$
$A_m$	$\tilde{{\tilde{T}}}_{m1}$	$\tilde{{\tilde{T}}}_{m2}$	$\ldots$	$\tilde{{\tilde{T}}}_{mm}$

**Table 2:** Linguistic scale for the HFAHP with the THFNs.

Linguistic term	THFNs
Absolutely high importance	$\tilde{{\tilde{9}}}= \lbrace (L_{9}^{\sigma (1)},9,U_{9}^{\sigma (1)}), \ldots , (L_{9}^{\sigma (p)},9,U_{9}^{\sigma (p)}) \rbrace$
Very high importance	$\tilde{{\tilde{8}}}= \lbrace (L_{8}^{\sigma (1)},8,U_{8}^{\sigma (1)}), \ldots , (L_{8}^{\sigma (p)},8,U_{8}^{\sigma (p)}) \rbrace$
high importance	$\tilde{{\tilde{7}}}= \lbrace (L_{7}^{\sigma (1)},7,U_{7}^{\sigma (1)}), \ldots , (L_{7}^{\sigma (p)},7,U_{7}^{\sigma (p)}) \rbrace$
Essential importance	$\tilde{{\tilde{6}}}= \lbrace (L_{6}^{\sigma (1)},6,U_{6}^{\sigma (1)}), \ldots , (L_{6}^{\sigma (p)},6,U_{6}^{\sigma (p)}) \rbrace$
Medium importance	$\tilde{{\tilde{5}}}= \lbrace (L_{5}^{\sigma (1)},5,U_{5}^{\sigma (1)}), \ldots , (L_{5}^{\sigma (p)},5,U_{5}^{\sigma (p)}) \rbrace$
Low importance	$\tilde{{\tilde{4}}}= \lbrace (L_{4}^{\sigma (1)},4,U_{4}^{\sigma (1)}), \ldots , (L_{4}^{\sigma (p)},4,U_{4}^{\sigma (p)}) \rbrace$
Very low importance	$\tilde{{\tilde{3}}}= \lbrace (L_{3}^{\sigma (1)},3,U_{3}^{\sigma (1)}), \ldots , (L_{3}^{\sigma (p)},3,U_{3}^{\sigma (p)}) \rbrace$
Absolutely low importance	$\tilde{{\tilde{2}}}= \lbrace (L_{2}^{\sigma (1)},2,U_{2}^{\sigma (1)}), \ldots , (L_{2}^{\sigma (p)},2,U_{2}^{\sigma (p)}) \rbrace$
Exactly equal	$\tilde{{\tilde{1}}}= \lbrace (1,1,1) \rbrace$

Step 1: The experts determine the relative importance of each pair in pairwise comparisons matrix with THFNs. Table 1 shows the HFPCM of performance of *n* alternatives.
  Where, $\tilde{{\tilde{T}}}_{ij}=\lbrace {\tilde{T}}_{ij}^{\sigma (l)} \rbrace _{l=1}^{p}$ represents the evaluations of *p* experts on comparison of *i*-th element to *j*-th element in a hesitant environment as THFN. It should be noted, since for each $i= 1,2,\ldots ,m$, importance of $A_i$ over $A_i$ is exactly equal, then $\tilde{{\tilde{T}}}_{ii}=\lbrace (1,1,1) \rbrace$. Table 2 shows the linguistic terms that are transformed into THFNs.
  For example, in Table 2
  $$\begin{aligned} \tilde{{\tilde{T}}}_{ij}=\tilde{{\tilde{k}}}= \lbrace (L_{k}^{\sigma (1)},k,U_{k}^{\sigma (1)}), \ldots , (L_{k}^{\sigma (p)},k,U_{k}^{\sigma (p)}) \rbrace \end{aligned}$$
  is representative worth of element *i* over element *j* under evaluation of *p* experts, which $L_{k}^{\sigma (l)}$ and $U_{k}^{\sigma (l)}$ for $l= 1, \ldots , p$ represent a fuzzy degree of *l*-th expert, if value of $U_{k}^{\sigma (l)} - L_{k}^{\sigma (l)}$ be greater, then it show more uncertainty in the opinion of the *l*-th expert. Note, if $\tilde{{\tilde{T}}}_{ij}$ is representative worth of element *i* over element *j*, then $\tilde{{\tilde{T}}}_{ji}=(\tilde{{\tilde{T}}}_{ij})^{-1}$.
Step 2: After formation HFPCMs with HFNs in Step 1, in this step we examine the acceptability of consistency of them by using corollary of Theorem 1.
Step 3: In this step, the amount of hesitant fuzzy synthetic extent for the *i*-th object of the HFPCM $[\tilde{{\tilde{T}}}_{ij}]_{m \times m}$, is obtained as
  5 $$\begin{aligned} \tilde{{\tilde{S}}}_{i}=\sum _{j=1}^{m}\tilde{{\tilde{T}}}_{ij} \mathop {\otimes }\limits _{\tilde{\tilde{}}} \bigg ( \sum _{i=1}^{m}\sum _{j=1}^{m}\tilde{{\tilde{T}}}_{ij} \bigg )^{-1} \end{aligned}$$
  where $\sum _{j=1}^{m}\tilde{{\tilde{T}}}_{ij}$ and $\bigg ( \sum _{i=1}^{m}\sum _{j=1}^{m}\tilde{{\tilde{T}}}_{ij} \bigg )^{-1}$ are THFNs, which are obtained as follows:
  $$\begin{aligned} \sum _{j=1}^{m}\tilde{{\tilde{T}}}_{ij}= \tilde{{\tilde{T}}}_{i1} \mathop {\oplus }\limits _{\tilde{\tilde{}}} \ldots \mathop {\oplus }\limits _{\tilde{\tilde{}}} \tilde{{\tilde{T}}}_{im} \end{aligned}$$
  and
  $$\begin{aligned} \bigg ( \sum _{i=1}^{m}\sum _{j=1}^{m}\tilde{{\tilde{T}}}_{ij} \bigg )^{-1}= \bigg (\tilde{{\tilde{T}}}_{11} \mathop {\oplus }\limits _{\tilde{\tilde{}}} \ldots \mathop {\oplus }\limits _{\tilde{\tilde{}}} \tilde{{\tilde{T}}}_{1m}, \ldots , \tilde{{\tilde{T}}}_{m1} \mathop {\oplus }\limits _{\tilde{\tilde{}}} \ldots \mathop {\oplus }\limits _{\tilde{\tilde{}}} \tilde{{\tilde{T}}}_{mm} \bigg )^{-1}. \end{aligned}$$
  Then, based on we assign weight of *i*-th agent in the HFPCM $\tilde{\tilde{{\textbf {A}}}}$ as follows:
  6 $$\begin{aligned} w_i=\min _{j} \lbrace h(\tilde{{\tilde{S}}}_i \ge \tilde{{\tilde{S}}}_j) \rbrace , \end{aligned}$$
  for each $j=1, \ldots , m$ and $j\ne i$. It should be noted that the following three conditions are considered to determine $h(\tilde{{\tilde{S}}}_i \ge \tilde{{\tilde{S}}}_j)$:
  i) If $S_i \ge S_j$ then $h^{\sigma (1)}(\tilde{{\tilde{S}}}_i \ge \tilde{{\tilde{S}}}_j)=1$, for each $l \in \lbrace 1, \ldots ,p \rbrace$.
  ii) If $L_{S_j}^{\sigma (1)} \ge U_{S_i}^{\sigma (l)}$ then $h^{\sigma (1)}(\tilde{{\tilde{S}}}_i \ge \tilde{{\tilde{S}}}_j)=0$.
  iii) In otherwise we have
    $$\begin{aligned} h^{\sigma (l)}(\tilde{{\tilde{S}}}_j \ge \tilde{{\tilde{S}}}_i)=\dfrac{L_{S_i}^{l} - U_{S_j}^{l}}{(S_j - U_{S_j}^{l})-(S_i - L_{S_i}^{l})}. \end{aligned}$$
Step 4: In this step, we take the hesitant fuzzy performance score of each alternative. For this purpose we use operations which are introduced in^76^ on HFEs. Based on, we can aggregate evaluations of all the experts by score functions in Definition 3 as a crisp decision-making.

## Illustration

For the verification of the proposed HFAHP algorithm one example is selected.

### Example 2

In a university assume that the post of a professor is vacant, and three volunteers $V_1, V_2$ and $V_3$ remain. A committee has convened to choose the best possible volunteer for the vacant post. The committee has two members and they have identified the following attributes for this selection:

- Educational capabilities (*E*)
- Research capabilities (*R*)
- Creativity implementation (*C*)
- Human maturity(*H*)

Table 3 presents, the HFPCM of performance attributes and Tables 4, 5, 6, 7 show the HFPCMs for the volunteers due to the attributes assigned by two experts in the form of THFNs. Finally, the goal is to select the best possible volunteer from the available options.

**Table 3:** HFPCM for attributes.

$\tilde{\tilde{{\textbf {B}}}}$	*E*	*R*	*C*	*H*
*E*	$\tilde{{\tilde{1}}}=\big \lbrace (1,1,1) \big \rbrace$	$\tilde{{\tilde{3}}}=\big \lbrace (2,3,4), (1,3,5) \big \rbrace$	$\tilde{{\tilde{1}}}=\big \lbrace (\frac{1}{2},1,\frac{3}{2}), (1,1,2) \big \rbrace$	$\tilde{{\tilde{2}}}=\big \lbrace (\frac{3}{2},2,\frac{5}{2}), (1,2,4) \big \rbrace$
*R*	$\tilde{\tilde{\frac{1}{3}}}=\big \lbrace (\frac{1}{4},\frac{1}{3},\frac{1}{2}), (\frac{1}{5},\frac{1}{3},1) \big \rbrace$	$\tilde{{\tilde{1}}}=\big \lbrace (1,1,1) \big \rbrace$	$\tilde{\tilde{\frac{1}{2}}}=\big \lbrace (\frac{2}{5},\frac{1}{2},1), (\frac{2}{7},\frac{1}{2},2) \big \rbrace$	$\tilde{\tilde{\frac{1}{4}}}=\big \lbrace (\frac{1}{5},\frac{1}{4},\frac{1}{3}), (\frac{1}{7},\frac{1}{4},\frac{1}{2}) \big \rbrace$
*C*	$\tilde{{\tilde{1}}}=\big \lbrace (\frac{1}{2},1,1), (\frac{2}{3},1,2) \big \rbrace$	$\tilde{{\tilde{2}}}=\big \lbrace (1,2,\frac{5}{2}), (\frac{1}{2},2,\frac{7}{2}) \big \rbrace$	$\tilde{{\tilde{1}}}=\big \lbrace (1,1,1) \big \rbrace$	$\tilde{\tilde{\frac{1}{2}}}=\big \lbrace (\frac{1}{3},\frac{1}{2},\frac{4}{3}) \big \rbrace$
*H*	$\tilde{\tilde{\frac{1}{2}}}=\big \lbrace (\frac{2}{5},\frac{1}{2},\frac{2}{3}), (\frac{1}{4},\frac{1}{2},1) \big \rbrace$	$\tilde{{\tilde{4}}}=\big \lbrace (3,4,5), (2,4,7) \big \rbrace$	$\tilde{{\tilde{2}}}=\big \lbrace (\frac{3}{4},2,3) \big \rbrace$	$\tilde{{\tilde{1}}}=\big \lbrace (1,1,1) \big \rbrace$

**Table 4:** HFPCM for volunteers with respect to *E*.

$\tilde{\tilde{{\textbf {E}}}}$	$V_1$	$V_2$	$V_3$
$V_1$	$\tilde{{\tilde{1}}}=\big \lbrace (1,1,1) \big \rbrace$	$\tilde{{\tilde{3}}}=\big \lbrace (\frac{3}{2},3,4), (1,3,5) \big \rbrace$	$\tilde{\tilde{\frac{1}{3}}}=\big \lbrace (\frac{1}{4},\frac{1}{3},\frac{1}{2}), (\frac{1}{5},\frac{1}{3},1) \big \rbrace$
$V_2$	$\tilde{\tilde{\frac{1}{3}}}=\big \lbrace (\frac{1}{4},\frac{1}{3},\frac{2}{3}), (\frac{1}{5},\frac{1}{3},1) \big \rbrace$	$\tilde{{\tilde{1}}}=\big \lbrace (1,1,1) \big \rbrace$	$\tilde{\tilde{\frac{1}{7}}}=\big \lbrace (\frac{1}{9},\frac{1}{7},\frac{1}{3}), (\frac{2}{17},\frac{1}{7},\frac{1}{2}) \big \rbrace$
$V_3$	$\tilde{{\tilde{3}}}=\big \lbrace (2,3,4), (1,3,5) \big \rbrace$	$\tilde{{\tilde{7}}}=\big \lbrace (2,7,\frac{17}{2}),(3,7,9) \big \rbrace$	$\tilde{{\tilde{1}}}=\big \lbrace (1,1,1) \big \rbrace$

**Table 5:** HFPCM for volunteers with respect to *R*.

$\tilde{\tilde{{\textbf {R}}}}$	$V_1$	$V_2$	$V_3$
$V_1$	$\tilde{{\tilde{1}}}=\big \lbrace (1,1,1) \big \rbrace$	$\tilde{{\tilde{3}}}=\big \lbrace (2,3,4), (\frac{3}{2},3,5) \big \rbrace$	$\tilde{{\tilde{3}}}=\big \lbrace (2,3,5) \big \rbrace$
$V_2$	$\tilde{\tilde{\frac{1}{3}}}=\big \lbrace (\frac{1}{4},\frac{1}{3},\frac{1}{2}), (\frac{1}{5},\frac{1}{3},\frac{2}{3}) \big \rbrace$	$\tilde{{\tilde{1}}}=\big \lbrace (1,1,1) \big \rbrace$	$\tilde{{\tilde{1}}}=\big \lbrace (1,1,1), (\frac{1}{2},1,2) \big \rbrace$
$V_3$	$\tilde{\tilde{\frac{1}{3}}}=\big \lbrace (\frac{1}{5},\frac{1}{3},\frac{1}{2}) \big \rbrace$	$\tilde{{\tilde{1}}}=\big \lbrace (1,1,1), (\frac{1}{2},1,2) \big \rbrace$	$\tilde{{\tilde{1}}}=\big \lbrace (1,1,1) \big \rbrace$

**Table 6:** HFPCM for volunteers with respect to *C*.

$\tilde{\tilde{{\textbf {C}}}}$	$V_1$	$V_2$	$V_3$
$V_1$	$\tilde{{\tilde{1}}}=\big \lbrace (1,1,1) \big \rbrace$	$\tilde{\tilde{\frac{1}{2}}}=\big \lbrace (\frac{1}{4},\frac{1}{2},1), (\frac{1}{5},\frac{1}{2},2) \big \rbrace$	$\tilde{{\tilde{3}}}=\big \lbrace (\frac{1}{2},3,4) \big \rbrace$
$V_2$	$\tilde{{\tilde{2}}}=\big \lbrace (1,2,4), (\frac{1}{2},2,5) \big \rbrace$	$\tilde{{\tilde{1}}}=\big \lbrace (1,1,1) \big \rbrace$	$\tilde{{\tilde{7}}}=\big \lbrace (3,7,8), (5,7,9) \big \rbrace$
$V_3$	$\tilde{\tilde{\frac{1}{3}}}=\big \lbrace (\frac{1}{4},\frac{1}{3},2) \big \rbrace$	$\tilde{\tilde{\frac{1}{7}}}=\big \lbrace (\frac{1}{9},\frac{1}{7},\frac{1}{5}), (\frac{1}{8},\frac{1}{7},\frac{1}{3}) \big \rbrace$	$\tilde{{\tilde{1}}}=\big \lbrace (1,1,1) \big \rbrace$

**Table 7:** HFPCM for volunteers with respect to *H*.

$\tilde{\tilde{{\textbf {H}}}}$	$V_1$	$V_2$	$V_3$
$V_1$	$\tilde{{\tilde{1}}}=\big \lbrace (1,1,1) \big \rbrace$	$\tilde{{\tilde{3}}}=\big \lbrace (2,3,4), (\frac{3}{2},3,5) \big \rbrace$	$\tilde{\tilde{\frac{1}{2}}}=\big \lbrace (\frac{2}{5},\frac{1}{2},\frac{2}{3}),(\frac{1}{4},\frac{1}{2},1) \big \rbrace$
$V_2$	$\tilde{\tilde{\frac{1}{3}}}=\big \lbrace (\frac{1}{4},\frac{1}{3},\frac{1}{2}), (\frac{1}{5},\frac{1}{3},\frac{2}{3}) \big \rbrace$	$\tilde{{\tilde{1}}}=\big \lbrace (1,1,1) \big \rbrace$	$\tilde{\tilde{\frac{1}{3}}}=\big \lbrace (\frac{2}{7},\frac{1}{3},\frac{2}{5}), (\frac{1}{5},\frac{1}{3},1) \big \rbrace$
$V_3$	$\tilde{{\tilde{2}}}=\big \lbrace (\frac{3}{2},2,\frac{5}{2}), (1,2,4) \big \rbrace$	$\tilde{{\tilde{3}}}=\big \lbrace (\frac{5}{2},3,\frac{7}{2}), (1,3,5) \big \rbrace$	$\tilde{{\tilde{1}}}=\big \lbrace (1,1,1) \big \rbrace$

First, we know that consistency of HFPCMs (see Tables 3, 4, 5, 6, 7) by using corollary of Theorem 1 is acceptable. Then due to Step 3, we obtain the weight vector with respect to the attributes and the weights of each volunteer under each attribute separately. For example, for weight vector with respect to the attributes, by using formula (5), we have

$$\begin{aligned} \tilde{{\tilde{S}}}_{E}= & {} \big \lbrace (0.1818,0.3320,0.6091), (0.1106,0.3320,0.9848)\big \rbrace \\ \tilde{{\tilde{S}}}_{R}= & {} \big \lbrace (0.0652,0.0988,0.2030), (0.0446,0.0988,0.3556)\big \rbrace \\ \tilde{{\tilde{S}}}_{C}= & {} \big \lbrace (0.1030,0.2134,0.3948), (0.0691,0.2134,0.6428)\big \rbrace \\ \tilde{{\tilde{S}}}_{H}= & {} \big \lbrace (0.1873,0.3557,0.6517), (0.1106,0.3557,0.9848)\big \rbrace \end{aligned}$$

Then, based on we assign weight of *i*-th agent in the HFPCMs in formula (6). The results are given in Tables 8 and 9.

**Table 8:** Hesitant fuzzy normalized weights of attribute.

Criterion	*E*	*R*	*C*	*H*
Weights	$\lbrace 0.37,0.30 \rbrace$	$\lbrace 0.02,0.15 \rbrace$	$\lbrace 0.23,0.24 \rbrace$	$\lbrace 0.38,0.31 \rbrace$

**Table 9:** Hesitant fuzzy normalized weights of volunteers under each attribute.

Criterion $\setminus$ Volunteers	$V_1$	$V_2$	$V_3$
*E*	$\lbrace 0.32,0.35 \rbrace$	$\lbrace 0,0.08 \rbrace$	$\lbrace 0.68,0.57 \rbrace$
*R*	$\lbrace 1,0.59 \rbrace$	$\lbrace 0,0.21 \rbrace$	$\lbrace 0,0.19 \rbrace$
*C*	$\lbrace 0.33,0.33 \rbrace$	$\lbrace 0.54,0.58 \rbrace$	$\lbrace 0.13,0.09 \rbrace$
*H*	$\lbrace 0.39,0.39 \rbrace$	$\lbrace 0,0.17 \rbrace$	$\lbrace 0.61,0.44 \rbrace$

Next we obtain the hesitant fuzzy performance score of each volunteer due to Step 4 as follows:

$$h_{\tilde{{\tilde{V}}}_{1}}=\lbrace 0.37,0.30 \rbrace \otimes \lbrace 0.32,0.35 \rbrace \oplus \lbrace 0.02,0.15 \rbrace \otimes \lbrace 1,0.59 \rbrace \oplus \lbrace 0.23,0.24 \rbrace \otimes \lbrace 0.33,0.33 \rbrace \oplus \lbrace 0.38,0.31 \rbrace \otimes \lbrace 0.39,0.39 \rbrace = \lbrace 0.32,0.32,0.30 \ldots 0.36,0.34,0.34 \rbrace _{1 \times 256}$$

$$h_{\tilde{{\tilde{V}}}_{2}}=\lbrace 0.37,0.30 \rbrace \otimes \lbrace 0.00,0.08 \rbrace \oplus \lbrace 0.02,0.15 \rbrace \otimes \lbrace 0.00,0.21 \rbrace \oplus \lbrace 0.23,0.24 \rbrace \otimes \lbrace 0.54,0.58 \rbrace \oplus \lbrace 0.38,0.31 \rbrace \otimes \lbrace 0.00,0.17 \rbrace = \lbrace 0.12,0.18,0.12 \ldots ,0.24,0.19,0.23 \rbrace _{1 \times 256}$$

$$h_{\tilde{{\tilde{V}}}_{3}}=\lbrace 0.37,0.30 \rbrace \otimes \lbrace 0.68,0.57 \rbrace \oplus \lbrace 0.02,0.15 \rbrace \otimes \lbrace 0.00,0.19 \rbrace \oplus \lbrace 0.23,0.24 \rbrace \otimes \lbrace 0.13,0.09 \rbrace \oplus \lbrace 0.38,0.31 \rbrace \otimes \lbrace 0.5,0.41 \rbrace = \lbrace 0.44,0.40,0.41 \ldots ,0.34,0.36,0.32 \rbrace _{1 \times 256}$$

## Comparative analysis

A common way to solve this group of problems is to aggregate the opinions of experts and convert them into the fuzzy or crisp problems, and then according to the available methods in the fuzzy and crisp space we solve these problems. In Example 2, if we use Change approach^7^, by taking the average value of experts opinion, we obtain Tables 10, 11, 12, 13, 14 as follows.

**Table 10:** The fuzzy evaluation matrix for attributes.

$\tilde{{\textbf {B}}}$	*E*	*R*	*C*	*H*
*E*	${\tilde{1}}= (1,1,1)$	${\tilde{3}}= (\frac{3}{2},3,\frac{9}{2})$	${\tilde{1}}= (\frac{3}{4},1,\frac{7}{4})$	${\tilde{2}}= (\frac{5}{4},2,\frac{13}{4})$
*R*	$\tilde{\frac{1}{3}}= (\frac{9}{40},\frac{1}{3},\frac{3}{4})$	${\tilde{1}}= (1,1,1)$	$\tilde{\frac{1}{2}}= (\frac{12}{35},\frac{1}{2},\frac{3}{2})$	$\tilde{\frac{1}{4}}= (\frac{6}{35},\frac{1}{4},\frac{5}{12})$
*C*	${\tilde{1}}= (\frac{7}{12},1,\frac{3}{2})$	${\tilde{2}}= (\frac{3}{4},2,3)$	${\tilde{1}}= (1,1,1)$	$\tilde{\frac{1}{2}}= (\frac{1}{3},\frac{1}{2},\frac{4}{3})$
*H*	$\tilde{\frac{1}{2}}= (\frac{13}{40},\frac{1}{2},\frac{5}{6})$	${\tilde{4}}= (\frac{5}{2},4,6)$	${\tilde{2}}= (\frac{3}{4},2,3)$	${\tilde{1}}= (1,1,1)$

**Table 11:** The fuzzy evaluation matrix for volunteers with respect to *E*.

$\tilde{{\textbf {E}}}$	$V_1$	$V_2$	$V_3$
$V_1$	${\tilde{1}}= (1,1,1)$	${\tilde{3}}= (\frac{5}{4},3,\frac{9}{2})$	$\tilde{\frac{1}{3}}= (\frac{9}{40},\frac{1}{3},\frac{3}{4})$
$V_2$	$\tilde{\frac{1}{3}}= (\frac{9}{40},\frac{1}{3},\frac{5}{6})$	${\tilde{1}}= (1,1,1)$	$\tilde{\frac{1}{7}}= (\frac{35}{306},\frac{1}{7},\frac{5}{12})$
$V_3$	${\tilde{3}}= (\frac{3}{2},3,\frac{9}{2})$	${\tilde{7}}= (\frac{5}{2},7,\frac{35}{4})$	${\tilde{1}}=(1,1,1)$

**Table 12:** The fuzzy evaluation matrix for volunteers with respect to *R*.

$\tilde{{\textbf {R}}}$	$V_1$	$V_2$	$V_3$
$V_1$	${\tilde{1}}= (1,1,1)$	${\tilde{3}}= (\frac{7}{4},3,\frac{9}{2})$	${\tilde{3}}= (2,3,5)$
$V_2$	$\tilde{\frac{1}{3}}= (\frac{9}{40},\frac{1}{3},\frac{7}{12})$	${\tilde{1}}= (1,1,1)$	${\tilde{1}}= (\frac{3}{4},1,\frac{3}{2})$
$V_3$	$\tilde{\frac{1}{3}}= (\frac{1}{5},\frac{1}{3},\frac{1}{2})$	${\tilde{1}}= (\frac{3}{4},1,\frac{3}{2})$	${\tilde{1}}= (1,1,1)$

**Table 13:** The fuzzy evaluation matrix for volunteers with respect to *C*.

$\tilde{{\textbf {C}}}$	$V_1$	$V_2$	$V_3$
$V_1$	${\tilde{1}}= (1,1,1)$	$\tilde{\frac{1}{2}}= (\frac{9}{40},\frac{1}{2},\frac{3}{2})$	${\tilde{3}}= (\frac{1}{2},3,4)$
$V_2$	${\tilde{2}}=(\frac{3}{4},2,\frac{9}{2})$	${\tilde{1}}= (1,1,1)$	${\tilde{7}}= (4,7,\frac{17}{2})$
$V_3$	$\tilde{\frac{1}{3}}= (\frac{1}{4},\frac{1}{3},2)$	$\tilde{\frac{1}{7}}= (\frac{17}{144},\frac{1}{7},\frac{4}{15})$	${\tilde{1}}= (1,1,1)$

Then, based on we assign weight of *i*-th agent (Table 14). The results are given in Tables 15 and 16.

**Table 14:** The fuzzy evaluation matrix for volunteers with respect to *H*.

$\tilde{{\textbf {H}}}$	$V_1$	$V_2$	$V_3$
$V_1$	${\tilde{1}}= (1,1,1)$	${\tilde{3}}= (\frac{7}{4},3,\frac{9}{2})$	$\tilde{\frac{1}{2}}= (\frac{13}{40},\frac{1}{2},\frac{5}{6})$
$V_2$	$\tilde{\frac{1}{3}}= (\frac{9}{40},\frac{1}{3},\frac{7}{12})$	${\tilde{1}}= (1,1,1)$	$\tilde{\frac{1}{3}}= (\frac{17}{70},\frac{1}{3},\frac{7}{10})$
$V_3$	${\tilde{2}}= (\frac{5}{4},2,\frac{13}{4})$	${\tilde{3}}= (\frac{7}{4},3,\frac{17}{4})$	${\tilde{1}}= (1,1,1)$

**Table 15:** Fuzzy normalized weights of attribute.

Criterion	*E*	*R*	*C*	*H*
Weights	0.32	0.11	0.24	0.33

**Table 16:** Fuzzy normalized weights of volunteers under each attribute.

Criterion $\setminus$ Volunteers	$V_1$	$V_2$	$V_3$
*E*	0.34	0.04	0.62
*R*	0.78	0.12	0.10
*C*	0.33	0.56	0.11
*H*	0.42	0.05	0.53

Finally, adding the weights per candidate multiplied by the weights of the corresponding criteria, a final score is obtained for each candidate. Table 17 shows a score for each volunteer by Change approach^7^ in the fuzzy environment.

**Table 17:** Final score of volunteers in fuzzy environment.

Volunteers	Final scores	Rank
$v_1$	0.41	1
$v_2$	0.18	2
$v_3$	0.41	1

As it can be seen that rank $v_1$ and $v_3$ are equal and their superiority over each other cannot be recognized. But if we use the proposed method for this problem without aggregate the experts’ opinions in the first step, the value of each option is in the form of a hesitant fuzzy set as shown in Table 18.

**Table 18:** Hesitant fuzzy score of volunteers by the new approach.

Volunteers	Hesitant fuzzy scores
$v_1$	$\lbrace 0.32,0.32,0.30 \ldots ,0.36,0.34,0.34 \rbrace _{1 \times 256}$
$v_2$	$\lbrace 0.12,0.18,0.12 \ldots ,0.24,0.19,0.23 \rbrace _{1 \times 256}$
$v_3$	$\lbrace 0.44,0.40,0.41 \ldots ,0.34,0.36,0.32 \rbrace _{1 \times 256}$

Now if we want to have a specific choice of volunteers, Table 19 shows a score for each volunteer by using defined score function in Definition 3.

**Table 19:** Final score of volunteers in hesitant fuzzy environment.

Volunteers	Final score	Rank
$v_1$	0.34	2
$v_2$	0.18	3
$v_3$	0.37	1

Tables 17 and 19 show that the final scores of the volunteers by evaluations of two members of committee in the new approach are different with aggregation in the fuzzy environment by Change approach^7^. Anyway, we can say that the results in a hesitant fuzzy approach are better than fuzzy approach, because in the fuzzy approach, in the first step aggregated information and we lost a lot of information and based on decision is made, but in the new approach until the last step, the calculations are performed in hesitant fuzzy space and we do not lose the problem information, except for the last step to determine the final score.

### Ethical approval

This article does not contain any studies with human participants or animals performed by any of the authors. Also, informed consent was obtained from all individual participants included in the study.

## Discussion

Decision problems are one of the issues that we face a lot in our daily life (such as buying a car, budget allocation, supply chain management, selecting top student and etc.). The existence of ambiguity in the opinion of the expert to determine the values of the criteria is one of the issues that makes the problem more complicated. The use of fuzzy sets is one of the solutions that can be considered for the effect of ambiguity in the problem. Many techniques and methods have been used to solve decision-making problems in fuzzy space. Now, if, in addition to ambiguity, the opinions of different experts are involved in decision-making, we will face a group decision-making problem in a fuzzy space. A common way to solve this group of problems is to aggregate the opinions of experts and convert it into a fuzzy or crisp problem, and then according to the available methods in the fuzzy and crisp space, it is done to solve this problem.

One of the problems in this method is that in the first stage of solving the problem, some of the information in the problem is lost by summarizing the opinions of the experts. In this paper, we have shown that the use of HFNs as a special case of HFSs can be of great help in solving group decision problems in fuzzy space and with the existence of arithmetic operators for HFNs, we can overcome these problems. In the example designed in section "Comparative analysis", we showed that the answer obtained in the proposed method is different and more useful than the answer obtained in the Change approach^7^, which can show the superiority of the new approach.

Of course, this method may have a series of bugs, such as a high calculation volume compared to existing methods for aggregating opinions in fuzzy or crisp form. But since it gives us more logical and acceptable answers, we can use it in problems that have high sensitivity in decision making. One of the most advantages of this approach can be to get of hesitant fuzzy scors. Also, in cases where decision-making is more sensitive and the use of methods with aggeregation opinions have no results, this new approach can be beneficial. It should be noted that the use of other types of fuzzy numbers can be used in the opinion of experts, but since this causes the complexity and high volume of calculations, we use THFNs that are very common and understandable. Generally, using of the HFNs and their applications in optimization and decision-making problems can be beneficial in maintaining problem information.

## Conclusion

This paper has shown how HFNs can be used to AHP method in the hesitant fuzzy environments. To solve this problem, at first we introduce a comparative method for two HFNs by extension principle on HFSs, then by it we investigated consistency in the HFPCMs. Finally we propose a new algorithm for HFAHP with THFNs that it gives a hesitant fuzzy performance score for ranking alternatives. It should be noted that due to the characteristics of HFNs and easier calculations on them, in future studies they can be used in other methods for AHP such as eigenvector, geometric mean and other decision-making methods in a hesitant fuzzy environments, in spite of some limitations they may have.

### Limitations of the proposed work

One of the limitations that can be considered for this approach, it is that the form of the some experts’ opinions may not be in the form of an HFN and provide as an HFS. Also, another limitation is to use the triangular HFNs form. Since it is common and effective to use triangular fuzzy numbers in fuzzy environments, we have considered this type of numbers for calculations. Although using other forms of HFNs can be used in this paper that it increases the volume and time of calculations and some definitions and formulas must be changed. However, the related challenges can be more, which we will address in future studies.

### Future work

In the future, we will be focusing on developing new approach for other methods in decision making such as TOPSIS, VIKOR, Best-Worth, ... that some of them may provide new definitions such as distance in this space. Also, the relationship between HFSs especially HFNs and other extensions of fuzzy sets such as intuitive fuzzy sets, neurosophic sets, soft sets, and the use of their combination in solving decision problems can be studied as the next future works.

## Data Availability

Data supporting this study are included within the article.
